# *Anaerobutyricum soehngenii* improves glycemic control and other markers of cardio-metabolic health in adults at risk of type 2 diabetes

**DOI:** 10.1080/19490976.2025.2504115

**Published:** 2025-05-15

**Authors:** Ilias Attaye, Julia K. Bird, Max Nieuwdorp, Sahin Gül, Jos F. M. L. Seegers, Steven Morrison, Stijn Hofkens, Hilde Herrema, Nam Bui, Marie-Luise Puhlmann, Willem M. de Vos

**Affiliations:** a Department of Internal and Vascular Medicine, Amsterdam University Medical Centers, The Netherlands; b Amsterdam Cardiovascular Sciences, Diabetes & Metabolism, Amsterdam, The Netherlands; c Bird Scientific Writing, Wassenaar, The Netherlands; d Department of Experimental Vascular Medicine, Amsterdam University Medical Centers, The Netherlands; e Caelus Health, Zegveld, The Netherlands; f P2F Ventures, Alton, UK; g Laboratory of Microbiology, Wageningen University, Wageningen, The Netherlands; h Human Microbiome Research Program, Faculty of Medicine, University of Helsinki, Finland

**Keywords:** *Anaerobutyricum*, next-gen beneficial microbes, butyrate-producing bacteria, prediabetes, type 2 diabetes, continuous glucose measurement, glycemic control, intestinal microbiota, responder analysis

## Abstract

*Anaerobutyricum soehngenii* (previously *Eubacterium hallii*) is a butyrate-producing next-generation beneficial microbe generally recognized as safe. Several short-term intervention trials by *A. soehngenii* L2–7 have shown improvement of insulin sensitivity in prediabetic subjects and type 2 diabetes patients. To determine the long-term cardiometabolic benefits and safety, we performed a 3-month double-blind, randomized placebo-controlled intervention in 98 prediabetic insulin-resistant adults in Europe and U.S. with daily administration of encapsulated cells of *A. soehngenii* CH-106, a tetracycline-sensitive isogenic derivative of strain L2–7. Compared to placebo, *A. soehngenii*-treated subjects showed significantly reduced glycemic variability (1% reduction in the coefficient of variation; *p* = 0.01) and improved glycemic control (6% reduction in the overall net glycemic action-1; *p* < 0.05), including reduced serum glycated hemoglobin (HbA1c) levels when including the 4-week washout period (1 mmol/mol reduction; *p* < 0.05). Moreover, diastolic blood pressure was significantly reduced in all *A. soehngenii*-treated subjects (3 mm Hg; *p* < 0.05). The study product was well-tolerated and had no effect on the global intestinal microbiota composition, including alpha and beta-diversity, besides an increased abundance of *A. soehngenii* in the treatment group, indicative of compliance. The U.S. participants, compared to those in Europe, responded best, notably in the oral glucose tolerance tests (15% improvement in the area-under-the curve of plasma glucose levels; *p* = 0.039) or coefficient of variation (reduction of 3.1%; *p* < 0.05). This potentially relates to a more severe prediabetic state in U.S. subjects, associated with significantly reduced (1.5–3.5-fold) relative abundance of *Bifidobacterium, Coprococcus*, *Ruminococcus* spp. and two-fold increased relative abundance of *Lachnoclostridium* spp. In conclusion, daily oral supplementation with *A. soehngenii* was safe and improved various markers of glycemic control, reduced HbA1c levels and diastolic blood pressure, indicating a novel microbiome-based approach to improve cardio-metabolic health in adults at risk for developing type 2 diabetes.

**Clinical trial reg. no.** NCT04529473, clinicaltrials.gov

**Social media summary 120 characters:** *Anaerobutyricum soehngenii* supplementation improves #cardio-metabolic health in subjects at risk for type 2 #diabetes

## GRAPHICAL ABSTRACT

**Figure:**
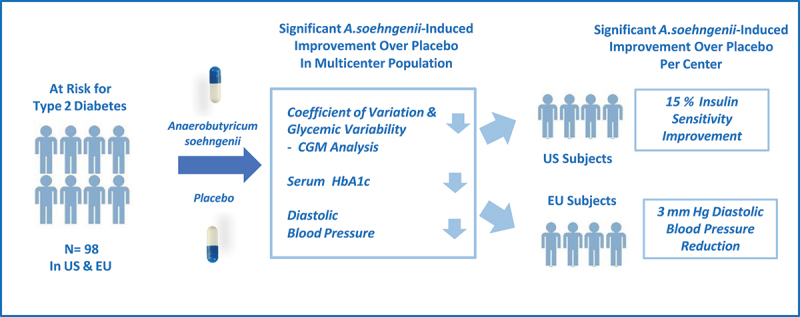

## Introduction

Type 2 diabetes is a chronic disease characterized by hyperglycemia and contributes considerably to global morbidity and mortality.^1^ Both the incidence and prevalence of type 2 diabetes have increased in recent decades due to rising obesity rates in many countries.^2^ Prediabetes is an insulin-resistant state in which blood glucose concentrations are increased, yet the affected subjects do not meet criteria for type 2 diabetes.^3^ Nevertheless, pathophysiological changes in tissues and organs related to hyperglycemia, including insulin resistance with hepatic glucose overproduction, beta-cell dysfunction, and low-grade inflammation also occur in prediabetic patients and contribute to macro- and microvascular complications. Hence, prediabetic subjects are at risk for developing type 2 diabetes. The global prevalence of prediabetes is increasing and around 9% of the adult population has impaired glucose tolerance, and 6% has impaired fasting glycemia, criteria used to define prediabetes.^4^

The intestinal microbiota has been recognized as a key driver of host health, although causal relationships have only been sparsely demonstrated.^5^ Intestinal microbiota deviations may play a role in the development of type 2 diabetes and several potential mechanisms have been investigated.^6^ The intestinal microbiota affects the permeability of the gastrointestinal mucosa, thus controlling the influx of inflammatory microbiota-derived components, such as lipopolysaccharides, into the circulation, which could drive chronic low-grade inflammation.^5^ Moreover, specific intestinal microbes convert both dietary and host components in the colon into short-chain fatty acids, notably propionate and butyrate, that all have intricate signaling capacities, resulting in improved intestinal barrier function.^5,7^ In early metagenomic studies of the gut microbes in type 2 diabetes patients, it was noted that butyrate-producing bacteria were depleted in comparison to healthy subjects.^8^ Hence, it has been proposed that increasing endogenous butyrate production by the gut microbiota could be used as a strategy for the prevention of obesity and related metabolic diseases.^9^ The supply of butyrate in the gut through microbiome modulation may be more effective than simple supplementation with butyrate, which showed metabolic improvement in animal models but not in adults with metabolic syndrome.^10^ One explanation is that oral supplementation with butyrate did not reduce lactate levels, which are known to be increased in insulin-resistant subjects.^11^

A recent deep metagenomic analysis of the gut microbiota in prediabetic subjects over time confirmed the importance of butyrate-producing bacteria, as several species were associated with improvement of metabolic syndrome, including *Coprococcus eutactus*, *Anaerostipes hadrus*, and *Eubacterium hallii*.^12^ Importantly, these intestinal species all have the capacity to convert lactate into propionate or butyrate. *Anaerobutyricum spp.*, previously known as *Eubacterium hallii*,^13^ are prevalent butyrate-producing members of the core intestinal microbiome in healthy subjects,^14^ already observed in the first months of life.^15^ The abundance of colonic *Anaerobutyricum spp*. was associated with insulin sensitivity in (pre)diabetic cohorts in the U.S. and China.^12,16^ Moreover, in the first trial demonstrating a causal relation between the gut microbiota and improvement of insulin sensitivity via duodenal fecal microbiota transfer, it was observed that the abundance of *Anaerobutyricum spp*. was increased in the upper intestinal tract of responders to this treatment.^17^

*Anaerobutyricum spp*. alone or in combination with *Bifidobacterium*, *Akkermansia spp.*, or synthetic minimal microbiomes have been found to produce butyrate via lactate and acetate derived from sugars, dietary polymers, and even mucin and human milk in trophic chains.^14,18,19^ Considerable attention has been given to *Anaerobutyricum soehngenii* strain L2–7, isolated from a healthy infant,^13^ which has been characterized by genomic, transcriptomic, and proteomic analysis in pure culture and synthetic communities.^14,20,21^ Likewise, its lactate-to-butyrate conversion has been subject to experimental analysis that showed the ability of *A. soehngenii* L2–7 to use both D-and L-isomers of lactate for generating butyrate.^20^ *A. soehngenii* strain L2–7 forms large (>10 μm long) cells^21^ and is known to deconjugate bile salts, detoxify toxic compounds, and produce antimicrobial compounds like 3-hydroxy-propionaldehyde (also known as reuterin) that may increase its intestinal competitiveness.^22,23^ In addition, also propionate can be directly produced by *A. soehngenii* L2–7 from 1,2-propanediol.^22^ This conversion is dependent on a vitamin B12 isomer that *A. soehngenii* L2–7 is capable of synthesizing and hence this strain can also drive trophic chains of intestinal microbes that use this vitamer to produce propionate.^19^

The effect of supplementing *A. soehngenii* strain L2–7 on insulin sensitivity has been investigated in various preclinical^24^ and clinical studies.^25,26^ A safety and proof-of-concept dose-finding trial in prediabetic subjects showed that *A. soehngenii* strain L2–7 administration for 4 weeks was well-tolerated. Moreover, significantly improved insulin sensitivity and blood pressure was observed in the high-dose intervention group.^25^ To address the mode of action, a single duodenal delivery of *A. soehngenii* strain L2–7 to metabolic syndrome subjects was found to improve glycemic control while increasing postprandial plasma GLP-1 levels, with a concomitant significant increase in small intestinal expression of the gene coding for regenerating islet protein 1B (Reg1B) synthesis.^26^ Additionally, secondary bile salts were increased upon this duodenal administration. Taken together, these results suggest that the site of action of orally administered *A. soehngenii* strain L2–7 is in the upper intestinal tract, where it induces GLP-1 excursion, potentially in response to butyrate or secondary bile salts.^27^ Recently, a third short-term (2 weeks) clinical trial was performed with *A. soehngenii* strain L2–7 in type 2 diabetes patients and showed reduced blood pressure and improvement of glycemic control even above the effect of the used metformin.^28^

In this study, we now expanded on previous results and examined the effect of a long-term (3 months) freeze-dried encapsulated daily administered placebo or *A. soehngenii* strain CH-106, a tetracycline-sensitive and isogenic derivative of strain L2–7, which we characterized here at the genomic level. *A. soehngenii* strain CH-106 has been subject to extensive characterization and toxicological testing^29^ and recently received, as one of the first next-generation beneficial microbes, a no objection letter from the U.S. Food and Drug Administration (FDA) related to its self-affirmed generally recognized as safe (GRAS) dossier.^30^ This study we describe here aimed to assess the safety and efficacy of *A. soehngenii* strain CH 106 on cardiometabolic health in adults at risk of type 2 diabetes in a 3-months randomized, parallel, placebo-controlled trial in North America (United States) and Europe (Ireland and United Kingdom). We show that this daily administration of *A. soehngenii* CH-106 significantly reduced glucose variability, decreased HbA1c levels, and lowered diastolic blood pressure compared to placebo. Moreover, microbiota and responder analysis revealed specific signatures that differentiated subjects in the studied continents and are likely to be involved in the response to the *A. soehngenii* intervention.

## Research design and methods

### Trial design and intervention

This trial was a multi-center, randomized, double-blind, placebo-controlled, parallel-group study to evaluate the effect of 12-weeks long daily oral supplementation with *A. soehngenii* CH-106 on glycemic control and insulin sensitivity in otherwise healthy hyperglycemic adults. Initially, the study was intended to be performed at one site in Ireland (Cork) and one site in the U.S. (Chicago, Illinois). Recruitment at a third site in Scotland (Glasgow) was added to increase recruitment. A sample size of 100 was based on an effect size of 0.25, an alpha of 0.05, power of 80% using repeated measures ANOVA and a two-sided effect. The study was conducted by Atlantia Clinical Trials, Cork, Ireland. The study had a double-blinded design and neither the investigators nor participants knew to which treatment group participants were assigned. The intervention consisted of one capsule per day containing 1 × 10^9^ active freeze-dried *A. soehngenii* strain CH-106. The placebo capsules consisted of identical capsules with maltodextrin.

Participants were randomized in a 1:1 ratio to either active intervention or placebo. Randomization was completed using a minimization procedure, which aimed to reduce imbalances in important baseline characteristics per site: BMI, Hb1Ac, and fasting blood glucose. Randomization was computer-generated. Randomization lists for the three sites were produced by a statistician not involved with the study and kept blinded in a separate, secure location at each site. The study product was dispensed in identical bottles, individually labeled with a subject randomization number that masked product allocation.

### Trial participants

Potential participants were recruited via the individual sites recruitment databases, advertisements on social media platforms, and local newspapers, as well as via general practitioners in the area close to the sites. The study population consisted of otherwise healthy adults, aged between 21 and 69 years with HbA1c between 5.5% (37 mmol/mol) and 8.0% (64 mmol/mol) with central obesity (waist circumference ≥94 cm). Female participants were aged between 45 and 69, and post-menopausal.

The trial was conducted according to the ethical principles presented in the Declaration of Helsinki as revised in 2013, the International Council for Harmonisation Guideline for Good Clinical Practice (ICH-GCP, E6 R2, November 2016), and all applicable local regulatory requirements. The clinical study protocol and accompanying material were approved by relevant independent ethics committees: for Ireland by the Clinical Research Ethics Committee of the Cork Teaching Hospitals and approved on 20th February 2020; for the U.S. site by Advarra IRB and approved on 14th February 2020; for Scotland by East of Scotland Research Ethics Service and approved on 25th November 2021. Written consent was obtained from all subjects prior to study-specific procedures. The study was conducted between July 2020 and August 2022 and completed as originally designed. The trial was registered at clinical trials gov (NCT04529473).

### Study procedures and endpoints

The study consisted of seven onsite visits over a study period of approximately 19 weeks (see Supplemental Table S1 for details). All primary and secondary endpoints were determined at the baseline and week 12 visits. In addition, the Continuous Glucose Monitoring (CGM) device (FreeStyle Libre Pro Flash Glucose Monitoring System) was applied 2 weeks before the baseline and week 12 visit. A stool sample was collected at baseline, week 12, and week 16 (for washout). HbA1c and fasting blood glucose samples were also taken at weeks 6 and 16 in addition to the baseline and week 12 visits. Safety was assessed by adverse event reporting at each site visit, monitoring of vital signs, and analyzing blood chemistry data.

Postprandial glucose and insulin excursion were determined as incremental area-under-the-curve (iAUC) derived from 2-h Oral Glucose Tolerance Test (OGTT). In short, subjects were fasted for a minimum of 8 h before the site visit, and an intravenous catheter was positioned in a distal arm vein. Blood was drawn at timepoint T −15, T0 min, T30, T60, T90, and T120 min. After T0 min patients received a standardized 75 g glucose to measure post-prandial glucose and insulin excursion. The iAUC was calculated using the trapezoidal method, ignoring the area below baseline, as previously described including the formula’s used.^31,32^ ISI-OGTT was calculated following the Matsuda formula described previously.^33^

All indices derived from the continuous glucose monitor (CGM) measurements, including the continuous overall net glycemic action (CONGA-1), the coefficient of variation (CV), and the time spent in the normal glucose range defined as 3.9–10.0 mmol/L, were determined via the CGDA R package (version 4.2.1).^34^ All relevant formulas and the complete R package can be found on GitHub (https://GitHub.com/EvdVossen/CGDA).

All blood chemical analysis was performed under supervision of Atlantia Clinical Trials, Cork Ireland, who coordinated the trial, using standard enzymatic, immune, and HPLC techniques in the U.S. by Labcorps and in Europe by Eurofins.

### A. soehngenii *CH-106: strain origin, genomic characterization, and preparation of the intervention product*

*A. soehngenii* CH-106 was obtained after ethyl methane sulfonate mutagenesis of its parental strain *A. soehngenii* L2–7 and selection for sensitivity to tetracycline.^29^ Apart from the selected tetracycline sensitivity, *A. soehngenii* CH-106 shows similar growth and butyrate production as the parental *A. soehngenii* L2–7 on sucrose, glucose, or lactate plus acetate.^20^ We also determined the complete genome sequence of *A. soehngenii* CH-106 using deep Illumina MiSeq sequencing and deposited this at the NCBI database under number PRJNA1219376 (coded R6M8). Comparison of the genomes of the isogenic *A. soehngenii* strains CH-106 and L2–7 (see PRJEB22345);^21^ showed that compared to the parental strain L2–7, strain CH-106 contained 37 mutations in the total genome of 3.5 Mb, 12 of which were in predicted non-coding regions and 25 in coding sequences, including 4 synonymous substitutions. The remaining 21 mutations included for the vast majority missense and nonsense mutations, 1 in frame 3-nucleotide deletion and 1 single nucleotide insertion. The latter insertion was located in the *tetO* gene and resulted in a frame-shift mutation leading to a truncated and hence inactive tetracycline resistance protein, explaining the sensitivity of *A. soehngenii* CH-106 to this antibiotic. Repeated growth experiments showed *A. soehngenii* CH-106 to be highly stable.

*A. soehngenii* strain CH-106^29^ was grown on a large scale on a sucrose-based medium, harvested, lyophilized, and mixed with maltodextrin.^27^ Approximately 350 mg of the resulting stable powder formulation was HACCP produced and packaged in Capsugel® DRcaps® (Lonza) with a concentration of 10^9^ Active Fluorescent Units (AFU) per capsule and used as a daily dose. Similarly made capsules containing an equivalent amount of maltodextrin only were used as a placebo. Participants were asked to store the product in the refrigerator. Before, during and after the trial, the number of active *A. soehngenii* cells was confirmed by determining the number of AFU. The AFU were determined using high-throughput flow cytometry analysis (FacsCanto II; BD Biosciences) according to ISO 19,344 of the International Dairy Federation.^35^

### Fecal sample collection and analysis

Fecal samples were collected by research participants at home, immediately frozen in a household freezer and brought in frozen state into the study site where they were stored at −80°C in the research lab where DNA was isolated, as previously recommended.^36^ DNA extraction from fecal samples was performed using QIAamp PowerFecal Pro DNA Kit according to the manufacturer’s instructions (Qiagen), including a bead-beating step that we have shown to be essential.^37^ DNA was quantified by fluorimetry with the Qubit dsDNA HS Assay Kit (Thermo Fisher Scientific).

Quantitative PCRs were performed in triplicate with Brilliant III Ultra-Fast SYBR Green QPCR Master Mix (Agilent) in a total volume of 10 μl with primers at 500 nM in 384-well plates sealed with optical sealing tape. Amplification was performed with a QuantStudio 12K Flex Real-Time PCR System (Applied Biosystems) and the data were analyzed using the QuantStudio 12K Flex Software. For quantification of total bacteria, Femto Bacterial DNA Quantification kit (Zymo Research) with 1 μL sample diluted 10,000-fold was used as a template.

For the detection of *A. soehngenii* CH-106, the strain-specific qPCR primers CHP-117 (5'ATGCCAGACGAGGATGAAGG) and CHP-118 (5’TCTCCTTCCGGCTTTCCTGT) described previously^24^ were used, and the concentrations of samples were calculated based on standard curves.

For 16S rRNA sequencing, the V3–V4 region of 16S rRNA gene was amplified using an adapted one-step PCR method on a Biometra Thermocycler at the Microbiota Center Amsterdam (Amsterdam UMC). Twenty nanograms of template DNA were used in the PCR mix, which contained 6 μL 5 × HF buffer (Thermo Fisher Scientific), 0.75 μL PCR Grade Nucleotide Mix (10 μM) (Thermo Fisher Scientific), 0.3 μL Phusion DNA Polymerase (2 U/µL), 18.95 μL nuclease-free water, 1.5 μl forward Index Primer (10 μM), and 1.5 μl reversed Index Primer (10 μM). The amplification program consisted of an initial denaturation at 98°C for 30 s, followed by 25 cycles of denaturation at 98°C for 10 s, annealing at 55°C for 20 s, elongation at 72°C for 90 s, and an extension at 72°C for 10 min. To confirm the presence of the PCR product, a 1% agarose gel electrophoresis containing ethidium bromide was used, and PCR products were purified using the Biomek FX robot with Ampure XP beads (Beckman Coulter). Amplicons were quantified using the Qubit® dsDNA BR Assay Kit. The samples were each labeled with a unique index and equimolar pooled. The pooled library was checked for quality and quantity using High Sensitive DNA chip (Agilent) on the Bioanalyzer 21,000 (Agilent) Qubit® dsDNA BR Assay Kit and sequenced by Illumina Miseq (v3, 600) sequencing. Raw sequence data were submitted at the European Nucleotide Archive (ENA) and are accessible at number PRJEB82846.

Amplicon sequences were parsed using a vsearch (v2.15.2) based pipeline. Paired-end reads were merged, with max differences set to 100 and allowing for staggered overlap. ASVs were inferred from reads with lower than 1.5 expected error rate using the cluster_unoise with centroids algorithm with a minsize of 4, after which chimeras were removed using the uchime3 denovo method. For each sample, ASV abundances were determined by mapping the merged reads against ASV sequences using usearch_global with a 0.97 distance cut off. Taxonomy was assigned using R (4.2.0) and the dada2 assign taxonomy function using the Silva (v132) reference database. A phylogenetic tree was generated use mafft (v7.310) and Fasttree (2.1.11).

### Statistical analysis

Statistical analysis was performed according to the principles of ICH-GCP. A Statistical Analysis Plan (SAP) was written prior to the first participant randomization and finalized prior to database lock. Intention-to-treat and per-protocol datasets were also defined prior to database lock. Only intention-to-treat analyses are described in this article.

Baseline measurements were analyzed using descriptive and inferential methods to determine if there were statistically significant baseline differences between treatment groups for the primary endpoints. Missing endpoints were handled statistically using available case analysis. If the Shapiro–Wilk’s test of normality was significant (*p* < 0.05), non-parametric testing was used to compare change in parameter per product (Mann–Whitney U-test). Data were also analyzed using linear mixed effects models, and delta changes were analyzed using linear regression models where appropriate. For the linear mixed effects models, the input was according to y = variable*group*center (fixed effects). Subject ID was used as random effect. For the delta changes, a linear regression model was used according to delta_change variable = group+baseline level variable. Analyses were performed on the total group and stratified per location. All analyses were corrected for site center when appropriate. Data are represented as median with interquartile ranges or means with standard deviation as indicated. A p-value <0.05 was considered statistically significant.

Statistical analyses were conducted using R Project for Statistical Computing Version 4.2.1. Specifically, package “tableone” (0.13.2) was used for descriptive statistics, linear mixed effects models were analyzed using “nlme” (3.1–163) and graphs were created with “ggplot2” (3.4.3). Continuous glucose monitor (CGM) data were analyzed by calculating summary metrics using the “CGDA” package.^34^ Microbiome analyses were conducted with the “vegan” package^38^ (25), the “phyloseq” package,^39^ the “microbiome” package,^40^ the “microViz” package,^41^ and the “mare” package.^38,42^

## Results

This study aimed to assess the safety and efficacy of once daily ingested capsules containing active cells of *A. soehngenii* strain CH-106 on cardiometabolic health in adults at risk of type 2 diabetes in a 3-months randomized, parallel, multicenter (Europe and U.S.) placebo-controlled trial. *A. soehngenii* strain CH-106 is an isogenic tetracycline-sensitive derivative of its parental strain *A. soehngenii* L2–7. Large-scale interventions and commercialization of antibiotic-resistant bacteria are not uncommon among bacteria that are marketed as probiotics.^43^ However, the use of probiotic strains with potentially transferable antibiotic resistance genes is considered unethical and unsafe.^44^ Hence, we developed *A. soehngenii* strain CH-106 for this trial and found it to be virtually identical to the parental *A. soehngenii* strain L2–7, except for the presence of an inactivated *tetO* gene, rendering strain CH-106 sensitive to tetracycline.

### Baseline data and compliance

The CONSORT flow chart depicts the flow of subjects through the study (Supplemental Figure S1). Overall, 98 subjects were included in the intention-to-treat population located in Europe (Cork IE and Glasgow UK) and the U.S. (Chicago). Baseline variables for demographic and anthropometric data as well as clinical endpoints showed no notable differences between treatment groups for the entire dataset (Table 1). However, several differences were noted between demographics and parameters at baseline when the two continents were compared. Compared to the recruited U.S. participants, those included at the European sites had higher age, a different ethnic pattern, and a lower proportion of any tertiary education, while they were more likely to be past smokers, and had a lower diastolic blood pressure. Related to the baseline values for prediabetic endpoints, the participants were characterized by higher insulin resistance (based on the OGTT results), body weight, and BMI, as well as higher serum levels of glycated hemoglobin (HbA1c), compared to the European participants. Overall compliance related to the study products (as determined by counting non-consumed capsules returned during return visits) was very good with 85% and 94% for placebo and *A. soehngenii* groups, respectively, consuming at least 80% of the study product over the intervention period.

**Table 1:** Baseline demographics and clinical endpoints.

		Entire dataset			Europe^†^			U.S.			p-value Europe vs. U.S.
Variable	Category	Ptylacebo	*A. soehngenii*	p-value	Placebo	*A. soehngenii*	p-value Europe	Placebo	*A. soehngenii*	p-value U.S.	
n		48	50		23	24		25	26		
Age (y)	Mean (SD)	51.8 (9.98)	53.2 (9.98)	0.462	53.1 (9.31)	57.5 (7.32)	0.077	50.52 (10.60)	49.31 (10.60)	0.685	**0.006**
Gender (M/F)	Female % (SD)	6 (12.5)	7 (14.0)	1	4 (17.4)	4 (16.7)	1	2 (8.0)	3 (11.5)	1	0.451
	Male % (SD)	42 (87.5)	43 (86.0)		19 (82.6)	20 (83.3)		23 (92.0)	23 (88.5)		
Ethnicity	Black % (SD)	9 (18.8)	10 (20.0)	0.986	1 (4.3)	0 (0.0)	0.102	8 (32.0)	10 (38.5)	0.417	**<0.001**
	Other % (SD)	7 (14.6)	7 (14.0)		3 (13.0)	0 (0.0)		4 (16.0)	7 (26.9)		
	White % (SD)	32 (66.7)	33 (66.0)		19 (82.6)	24 (100.0)		13 (52.0)	9 (34.6)		
Education	Any tertiary % (SD)	34 (70.8)	41 (82.0)	0.427	12 (52.2)	16 (66.7)	0.563	22 (88.0)	25 (96.2)	0.471	**0.001**
	Other % (SD)	3 (6.2)	2 (4.0)		2 (8.7)	2 (8.3)		1 (4.0)	0 (0.0)		
	Secondary completed % (SD)	11 (22.9)	7 (14.0)		9 (39.1)	6 (25.0)		2 (8.0)	1 (3.8)		
Smoking status	Current smoker % (SD)	6 (12.5)	4 (8.0)	0.745	2 (8.7)	3 (12.5)	0.868	4 (16.0)	1 (3.8)	0.313	**0.003**
	Non-smoker % (SD)	33 (68.8)	37 (74.0)		13 (56.5)	14 (58.3)		20 (80.0)	23 (88.5)		
	Past smoker % (SD)	9 (18.8)	9 (18.0)		8 (34.8)	7 (29.2)		1 (4.0)	2 (7.7)		
Alcohol consumption	Consumes alcohol % (SD)	38 (79.2)	41 (82.0)	0.921	18 (78.3)	20 (83.3)	0.943	20 (80.0)	21 (80.8)	1	1
	Does not consume alcohol % (SD)	10 (20.8)	9 (18.0)		5 (21.7)	4 (16.7)		5 (20.0)	5 (19.2)		
BMI (kg/m2)	Mean (SD)	33.04 (5.37)	31.56 (4.75)	0.151	31.84 (4.26)	30.14 (4.13)	0.172	34.14 (6.10)	32.86 (4.99)	0.418	**0.014**
Body weight (kg)	Mean (SD)	103.21 (20.50)	96.79 (16.38)	0.089	98.00 (17.00)	92.33 (14.72)	0.227	108.00 (22.55)	100.90 (17.02)	0.21	**0.013**
HbA1c (mol/mol)	Mean (SD)	40.85 (4.54)	41.20 (4.88)	0.712	40.65 (4.46)	39.25 (3.74)	0.248	41.03 (4.70)	43.00 (5.17)	0.16	**0.026**
Insulin Sensitivity (ISI) Index from OGTT	Mean (SD)	3.02 (2.44)	3.23 (2.44)	0.672	3.18 (2.98)	4.07 (2.81)	0.299	2.87 (1.85)	2.45 (1.76)	0.413	**0.046**
2 h blood glucose OGTT iAUC	Mean (SD)	357 (193.68)	355.71 (205.53)	0.983	385.91 (227.01)	318.02 (207.75)	0.295	330.80 (159.12)	390.49 (201.17)	0.247	0.792
2 h insulin OGTT iAUC	Mean (SD)	9233.12 (5174.04)	9048.92 (8539.02)	0.899	8751.95 (4932.99)	6573.27 (5538.40)	0.167	9656.56 (5442.32)	11334.14 (10167.52)	0.469	**0.043**
Fasting blood glucose	Mean (SD)	5.84 (1.11)	5.89 (0.69)	0.791	6.00 (1.44)	5.75 (0.58)	0.436	5.70 (0.69)	6.02 (0.76)	0.116	0.954
Systolic blood pressure	Mean (SD)	129.26 (11.18)	132.56 (12.88)	0.179	133.51 (9.37)	132.60 (14.45)	0.8	125.35 (11.45)	132.53 (11.53)	**0.03**	0.1
Diastolic blood pressure	Mean (SD)	86.06 (6.81)	84.62 (9.97)	0.407	86.14 (7.35)	80.60 (10.78)	**0.046**	85.99 (6.43)	88.33 (7.62)	0.241	**0.024**
Blood glucose variability: Continuous overall net glycemic action (CONGA-1)	Mean (SD)	1.19 (0.31)	1.23 (0.34)	0.514	1.25 (0.38)	1.27 (0.39)	0.898	1.14 (0.24)	1.21 (0.29)	0.356	0.204
Blood glucose control: % time in normal range	Mean (SD)	90.60 (17.37)	93.19 (10.72)	0.384	84.03 (23.42)	95.60 (4.33)	**0.028**	96.39 (4.88)	91.15 (13.81)	0.079	0.189

*The p-value for categorical variables p-value calculated from the Pearson’s chi-squared test for count data, and from an independent, two-sample t-test for continuous. variables. Bold text indicates significant differences (*p* < 0.05). ^†^The subjects in Cork, Ireland (20 receiving *A. soehngenii* and 19 placebo) and Glasgow, Scotland (4 receiving *A. soehngenii* and 4 placebo) were taken together in the analysis and labeled as European.

### Glycemic control and cardio-metabolic health parameters

#### 2-h OGTT and HbA1c results

**Figure 1.**
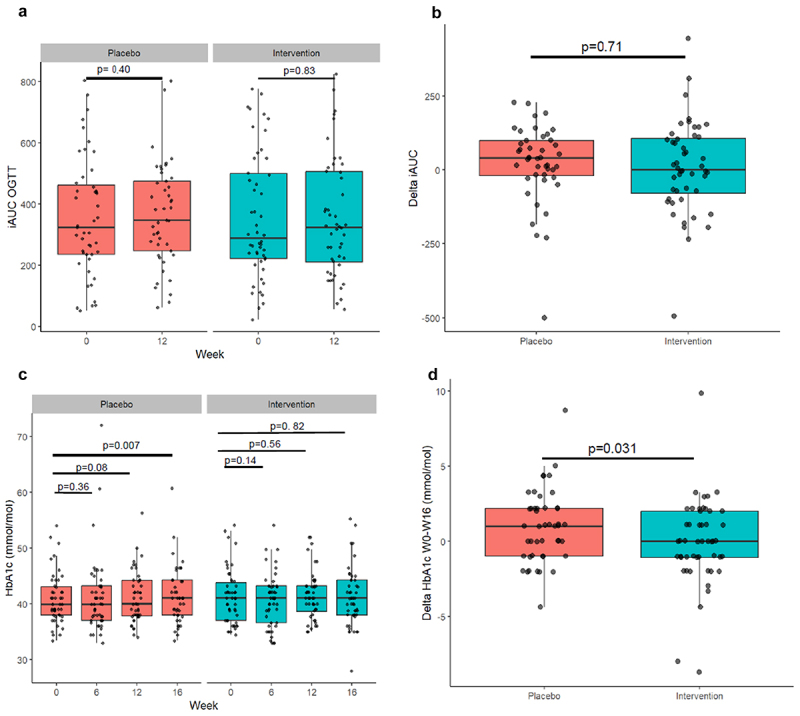
Effect of *A. soehngenii* supplementation on (a) iAUC after OGTT, (b) delta change iAUC, (c) HbA1c, and (d) delta HbA1c between week 0 and week 16. Analyses performed using a linear mixed effects model. *p* < 0.05 was considered statistically significant. Abbreviations used: IAUC =  incremental area under the curve; OGTT = Oral glucose tolerance test.

Glucose responses during OGTT were determined at baseline and after 12 weeks of intervention, whereas HbA1c concentrations were determined at regular intervals up to 16 weeks (Figure 1). We observed no significant change in the OGTT glucose iAUC within or between placebo and *A. soehngenii* supplementation groups (Figure 1a,b). However, when compared to placebo, HbA1c levels were significantly lower in the *A. soehngenii* group after 16 weeks (*p* = 0.031; Figure 1c,d). The decrease was 0.1% (1 mmol/mol) HbA1c from the average baseline level of 5.8% (40 mmol/mol; Table 1 and Supplemental Table S2).

The study site-wise analysis (Supplemental Figure S2A) subsequently showed that OGTT glucose iAUC increased in the placebo group and decreased in the *A. soehngenii* supplementation group in the U.S. with the difference (approximately 15% improvement) between both treatments being statistically significant (*p* = 0.039). Similarly, when comparing sites for the HbA1c levels after 16 weeks using a linear mixed-effects model (Supplemental Figure S2B), the U.S. subjects showed a slightly higher HbA1c improvement (1.1 mmol/mol) in the *A. soehngenii* group over placebo as compared to the total cohort improvement (*p* = 0.05; see also Supplemental Table S2).

#### Glycemic variability – coefficient of variation and CONGA-1 values

**Figure 2.**
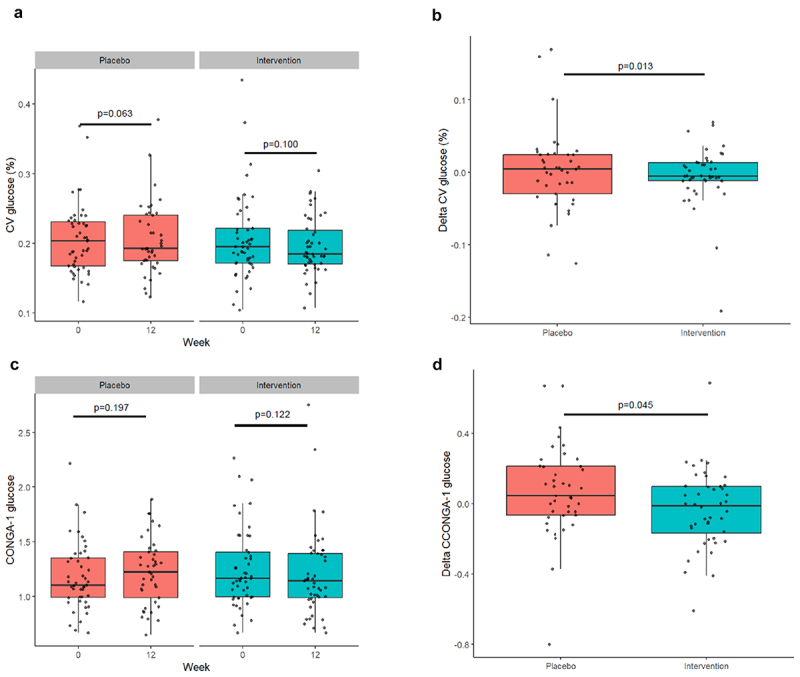
Effect of *A. soehngenii* supplementation on glycemic variability metrics. (a) Coefficient of variance (CV), (b) delta change CV, (c) continuous overall net glycemic action (CONGA), and (d) delta CONGA between. All analyses performed using a linear mixed effects model. *p* < 0.05 was considered statistically significant.

To provide a dynamic picture of whole-body glucose levels, we applied CGM in both placebo and *A. soehngenii* supplemented subjects (Figure 2). Although the coefficient of variation was not significantly altered compared to baseline in both groups (Figure 2a), we did observe a reduction in the coefficient of variation of glucose levels upon *A. soehngenii* supplementation of 1% when compared to placebo (*p* = 0.013; Figure 2b). Similarly, the net glycemic action as reflected in the Continuous Overlapping Net Glycemic Action (CONGA-1) values showed no significant reduction from baseline in both groups (Figure 2c). However, the decrease of overall change in CONGA-1 values (6%) was significant in the *A. soehngenii* supplementation group when compared to placebo (*p* = 0.045; Figure 2d). Finally, when we compared sites using linear mixed effects models (Supplemental Figures S2C and S2D), we observed significant reductions in glucose variability (CONGA-1) upon treatment notably in subjects in the U.S. (approximately 20% reduction; *p* = 0.032) and glycemic variation (4% reduction; *p* = 0.033) (see also Supplemental Table S2). However, the differences between the sites were small and showed conflicting directions for the European subjects in contrast to those in the U.S.

### Systolic and diastolic blood pressure

**Figure 3.**
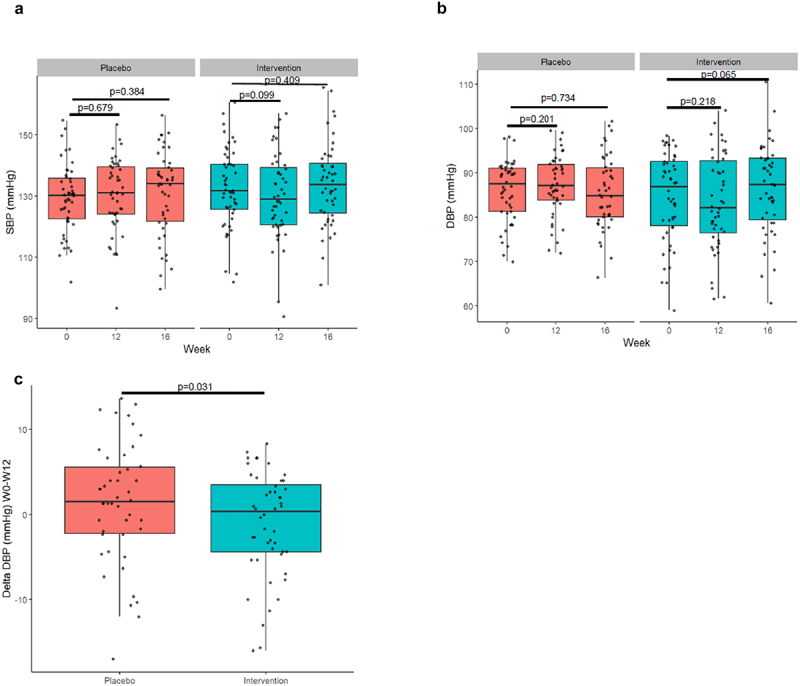
Effect of *A. soehngenii* supplementation on (a) systolic blood pressure, (b) diastolic blood pressure, and (c) change in diastolic blood pressure between week 0 and week 12. Delta analyses performed using a linear regression model on total dataset and linear mixed effects models for within group comparisons. *p* < 0.05 was considered statistically significant.

Office blood pressure measurements were taken at baseline, week 12, and after a 4-week washout in week 16. Overall, there were no significant changes in average blood pressure across timepoints using a linear regression model (Figure 3a,b). However, compared to baseline, both systolic and diastolic blood pressure did show a trend toward reduction after 12 weeks of *A. soehngenii* supplementation (*p* = 0.09 and *p* = 0.21, respectively). Diastolic blood pressure showed a significant decrease of approximately 1 mmHg in the *A. soehngenii* supplementation group when compared to placebo (*p* = 0.03; Figure 3c). Subsequent site-wise analysis showed that the diastolic blood pressure changes were significantly different (*p* = 0.002) and were reduced most in the European subjects, with a reduction of 3.8 mm Hg observed after 12 weeks supplementation of *A. soehngenii* compared to baseline (Supplemental Figures S2E and S2F; Supplemental Table S2). Of note, after the 4-week washout period (week 16), there were no significant differences in diastolic (or systolic) blood pressure.

### Safety summary

Daily oral supplementation of encapsulated freeze-dried *A. soehngenii* was well-tolerated during the study, with no serious adverse events (SAE) observed. This is in line with the observation that only two of the 51 subjects in the intervention group withdrew from the trial, with similar low numbers in the placebo group where also two from the 49 subjects dropped out (See Figure S1). All reported adverse events (AEs) were of mild or moderate intensity, and only six events in total were deemed to be related to the study product (three related to *A. soehngenii* and three to placebo; see Supplemental Table S3–4).

Laboratory results of safety blood biochemistry parameters were generally unaltered and remained within normal ranges at all study visits. Individual results were clinically reviewed by the sub-investigator and deemed to be safe at all timepoints. There were no clinically meaningful changes in participants’ anthropometrics or vital signs detected during the study.

### Fecal microbiota composition

**Figure 4.**
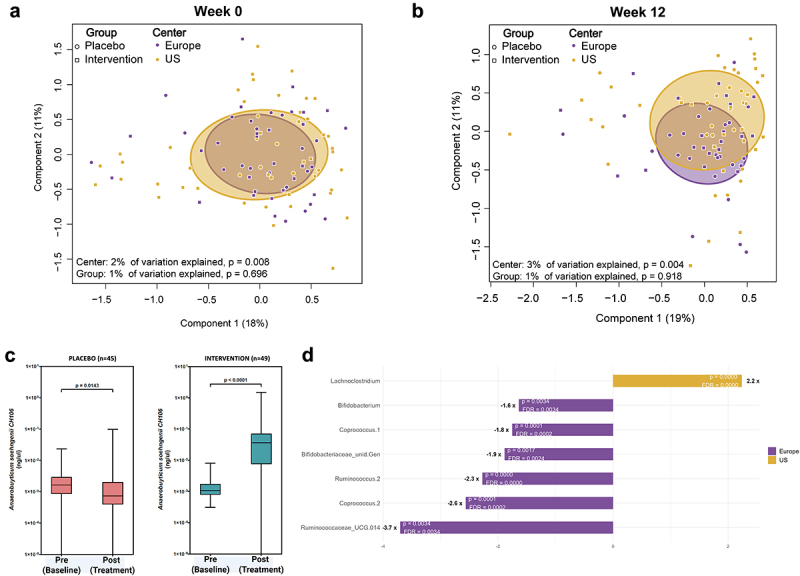
Gut microbiota analysis based on 16S rRNA amplicon sequencing and qPCR. The overall principal coordinate analysis (PCoA) of the microbiota composition (α-diversity based on Bray–Curtis dissimilarity) in the *A. soehngenii*-treated and placebo groups before (a) and after intervention (b) are shown, revealing significant center-based effects. (c) The ratio of *A. soehngenii* CH-106 rRNA over total bacterial rRNA at time points visit 3 (week 0 – baseline) and visit 6 (week 12 – end of the intervention). (d) The bacterial signature that differentiates the gut microbiota of the European (EU) and U.S. participants at baseline with relevant fold-change, p-value and false discovery rate (FDR).

The intestinal microbiota composition of subjects in the trial was determined by sequence analysis of 16S rRNA amplicons and qPCR analysis of fecal DNA (Figure 4). No significant differences between the global microbiota composition assessed by Principal Coordinate Analysis (PCoA based on β-diversity using Bray–Curtis dissimilarity) were observed at baseline and end of the trial in either the *A. soehngenii*-treated or placebo group (group difference explained less than 1% of variance; Figure 4a,b). In line with this, no differences between richness, evenness, and α–diversity were observed (Supplemental Table S5). The effect of the *A. soehngenii* treatment was more pronounced in the U.S. than in the European participants, likely because the latter were less obese or diabetic (see Table 1). Hence, we addressed the intestinal microbiota composition (PCoA of β-diversity based on Bray–Curtis dissimilarity) and found this to differ considerably between these two groups both at baseline (PERMANOVA *p* = 0.008) and at the end of the intervention (*p* = 0.004) (center: 2% or 3% of variance explained; Figure 4a,b, respectively).

The level of the *A. soehngenii*-derived amplicons was too low to be accurately established by the 16S rRNA sequencing approach. Hence, we determined the relative abundance of *A. soehngenii* CH-106 by qPCR and found this to increase close to a hundred-fold in the *A. soehngenii*-treated group from week 0 to week 12 (*p* < 0.001) (Figure 4c). This confirmed the compliance of the subjects to the intervention product and is in line with earlier studies where no effect on the global intestinal microbiota composition was observed of the intervention with *A. soehngenii* L2–7.^25,28^ Similarly, the absolute amount of *A. soehngenii* CH-106 as copies per gram of feces was determined by qPCR in both groups before and after the intervention, revealing a significantly (*p* < 0.0001) increased level of *A. soehngenii* CH-106 in the *A. soehngenii*-treated group (Supplemental Figure S3). In contrast, *A. soehngenii* CH-106 levels were decreased in the placebo group (*p* = 0.0143), in line with earlier observations that the abundance of *Anaerobutyricum* spp. decreased in a longitudinal analysis of prediabetic subjects^12^.

The clinical benefit of the *A. soehngenii* treatment was more pronounced in the U.S. than in the European participants (see Supplemental Table S2), likely because the latter were less obese and/or insulin resistant (see Table 1). Hence, we addressed the intestinal microbiota composition of the subjects in both continents and found this to differ considerably between these two groups both at baseline (*p* = 0.008) and at the end of the intervention (*p* = 0.004) (center: 2% or 3% of variance explained; Figure 4a,b, respectively). Detailed analysis revealed that the baseline intestinal microbiota of the U.S. participants compared to that of the European participants was characterized by a 2-fold higher relative abundance of *Lachnoclostridium* spp. and lower relative abundance (approximately 2-fold) of *Ruminococcus (*notably *R. bromii), Coprococcus*, and *Bifidobacterium* (notably *B. adolescentsi*) spp. (Figure 4d). These differences remained after the *A. soehngenii* intervention.

**Figure 5.**
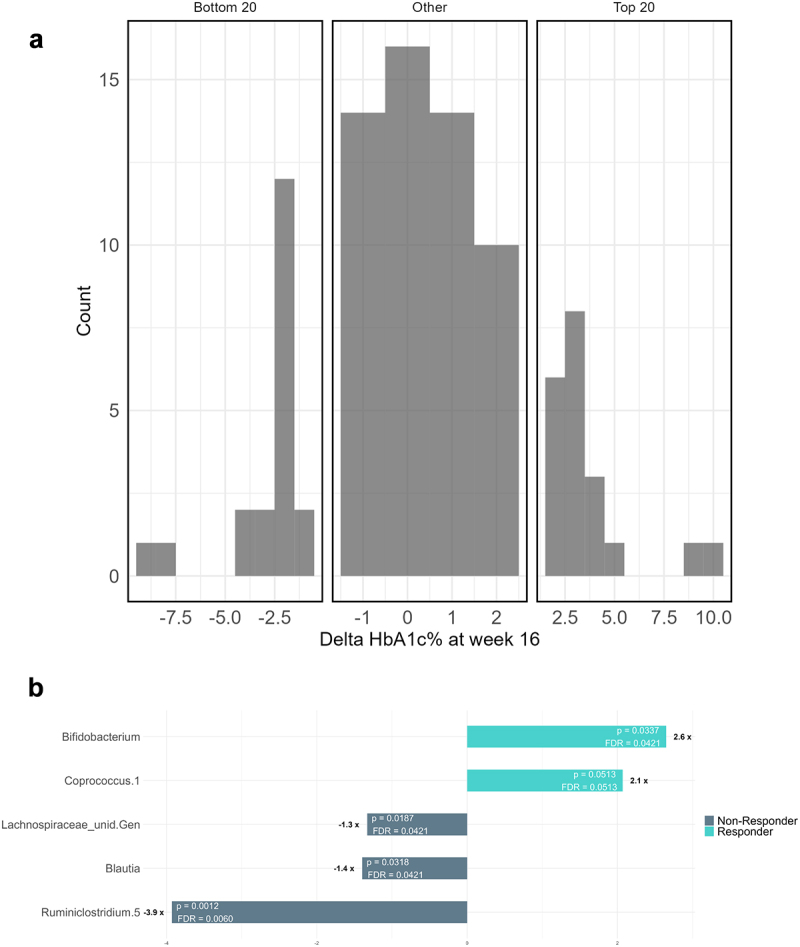
Distribution of the improvement in serum HbA1C over baseline in the intervention group (a). The signature with the most representative taxa that differentiate responders and non-responders is shown with relevant fold-change, p-value and false discovery rate (FDR) (b).

The most clinically relevant improvement observed in this trial was the reduction of serum HbA1c after the *A. soehngenii* intervention (Figure 1d). To study whether specific microbiota signatures could be associated with the reduction in HbA1c levels, we segmented the *A. soehngenii-*treated participants into a group of high responders that showed a high HbA1c level reduction (0.5–4 mmol/mol; *n* = 20) versus non-responders with no reduction in HbA1c levels (1–5 mmol/mol; *n* = 20) (Figure 5a). Comparison of the microbiota composition between these two groups revealed the high responders to be characterized at baseline by 2.5-fold higher relative levels of *Bifidobacterium* spp., 2-fold increased level of *Coprococcus* spp., slightly reduced levels of Unidentified *Lachnospiracaea* and *Blautia* spp., and almost 4-fold reduced levels of *Ruminoclostridium 5* spp. compared to non-responders (Figure 5b).

## Discussion

Encapsulated freeze-dried *A. soehngenii* CH-106, an isogenic tetracycline-sensitive derivative of *A. soehngenii* L2–7, was administered orally once daily for 3 months in European and U.S. subjects at risk for type 2 diabetes. This intervention significantly improved various markers of glycemic control, reduced serum HbA1c-levels over an extended period, and reduced diastolic blood pressure when compared to placebo – all previously set primary or secondary endpoints of the study (see NCT04529473). The study product was also well-tolerated (no serious adverse events) with the adverse event profile being similar between the placebo and *A. soehngenii*-treated groups.

The relative abundance of *A. soehngenii* increased considerably in the fecai samples of the intervention group, indicating that the study product was consumed and successfully passed the putative site of action in small intestine, subsequently reaching the colon (Figure 4c). Of note, the fecal abundance of *A. soehngenii* was found to decrease slightly in the placebo group (Figure 4c). This mimics earlier studies reporting the reduced relative abundance of *Anaerobutyricum* spp. in type 2 diabetes patients.^12,16^

Participants included in this study were otherwise healthy but prediabetic subjects with only slightly elevated fasting glucose levels. As these subjects are known to be at risk of developing type 2 diabetes, the purpose of the study was to improve glycemic control by supplementation with *A. soehngenii* as compared to placebo. Approximately half (*n* = 47) of the subjects were recruited in Europe (Cork and Glasgow), whereas the other half (*n* = 51) were from the U.S. (Chicago). The U.S. subjects were characterized by a more pronounced prediabetic state as evident from their baseline values (Table 1). *A. soehngenii* supplementation had the largest effect in the U.S. subjects, suggesting a therapeutic effect in further advanced stages of aberrant glucose control. Indeed, one of the clinical endpoints in this study was improvement on OGTT-stimulated glucose excursions, which showed in the U.S. population a statistically significant improvement of approximately 15% of glucose iAUC. Furthermore, it was found when including all subjects that *A. soehngenii* supplementation significantly reduced blood HbA1c levels by 0.1% (1 mmol/mol) when compared to placebo after 16 weeks, a period that included the 4-week washout period. This likely allowed for the effect of the intervention to become manifest since, generally, it takes several months to observe changes in serum HbA1c due to the 3–4 months lifespan of red blood cells. The observed serum HbA1c reduction upon *A. soehngenii* supplementation is similar to a decrease of %HbA1c, from 5.8% (40 mol/mol) to 5.6% (38 mol/mol), a small but clinically relevant improvement.^45^

The use of CGM enabled us to further dissect several components of glucose variability over time.^34^ Indeed, CGM data analyses showed that *A. soehngenii* supplementation significantly reduced glycemic variability. Many subjects had a coefficient of glucose variation of over 20%, which is considered undesirable, and the *A. soehngenii* supplementation reduced this to values that are acceptable for a healthy population.^46^ Similarly, glycemic control as determined by CONGA-1 was also found to improve by approximately 5% after *A. soehngenii* supplementation. Together, these CGM-derived parameters underscore the improvement in glucose variability and hence increased glycemic control by *A. soehngenii* in prediabetic subjects. Similarly, the clinical end points derived from the CGM data, including the coefficient of variation and CONGA-1, were significantly improved in the entire study-group but once again the largest effects (improvements of 4% and 20%, respectively) were observed in the U.S. subjects.

Of particular scientific and clinical interest was the observation that both systolic and diastolic blood pressure were lowered after *A. soehngenii* CH-106 supplementation, as previously observed with *A. soehngenii* L2–7^25^ but only the reduction in diastolic blood pressure reached significance. Of interest, the reduced blood pressure returned to baseline values in the wash-out phase, indicating the reversibility of this parameter and supporting the causal effect of the *A. soehngenii* CH-106 supplementation on this cardio-metabolic parameter. While the improvement of blood pressure was significant for diastolic blood pressure in the entire group, it was most notably seen in the European subjects with a reduction of 3.8 mm Hg after 12 weeks supplementation of *A. soehngenii* CH-106. This medically relevant effect^47^ could be driven by enhanced production of intestinal butyrate, which is known to reduce blood pressure in rodent models and has suggested to be a likely candidate to be effective in humans.^48^

The results of our current study are in agreement with earlier studies with *A. soehngenii* in metabolic syndrome subjects, demonstrating improvements in insulin sensitivity and reduction of blood pressure and addressing the mode of action.^25,26^ Moreover, our current study has improved robustness due to the larger sample size and the use of a placebo group, while complying with current clinical practices including the use of the state-of-the art CGM with a 12-week intervention and 4-week follow-up washout period with longitudinal sampling. All in all, this allowed to detect the worsening of glycemic control and Hb1Ac levels of the prediabetic subjects in the placebo group over time as has been previously observed in intervention trials in prediabetic subjects.^49^

Using 16S rRNA amplicon sequencing and qPCR analysis, we determined the treatment effect *A. soehngeni*i CH-106 on the gut microbiota composition. We observed no impact on the diversity measures or relative abundance of any taxa in the gut microbiota during the intervention apart from the increase in the abundance of the administered *A. soehngenii* strain CH-106 (See Figure 4). This is in line with an earlier intervention study with the parental *A. soehngenii* strain L2–7 where deep metagenomic analysis showed no impact on the community but an increased relative level and replication rate of *A. soehngenii*.^25^ The absence of global effects on the intestinal microbiota was independent of the site where the trial was performed. However, the baseline values of the subjects in Europe and U.S. were considerably different (Table 1), and this was associated with notable differences in the microbiota composition (Figure 4). The relative abundance of several taxa considered to be associated with health was significantly lower in the U.S. subjects as compared to those in Europe, in line with their more advanced prediabetic state (Table 1; Figure 4). These include reduced relative levels of *Bifidobacterium* spp., mostly consisting of *B. adolescentis* that are prevalent in adults and have reported beneficial properties,^50^ *Ruminococcus* spp. with the most prevalent *R. bromii* as a key stone species converting polymers, such as resistant starch into acetate that can be used by butyrate-producing bacteria,^51^ and *Coprococcus* spp., including butyrate-producers that were recently described as biomarkers for mental health.^52^ Of note, in comparison with the European subjects, those in the U.S. showed a 2-fold higher relative abundance of *Lachnoclostridium* spp., known to be capable of producing trimethylamine, a compound associated with cardiovascular risk.^53^

Part of the microbiota signature that was found in the less prediabetic subjects in Europe was also found in the subjects that responded the best to the *A. soehngenii* administration and improved the most in the HbA1c (Figure 5). Compared to the non-responders, these included a several-fold increased relative abundance of *Bifidobacterium* and *Coprococcus* spp. as well as an almost 4-fold reduced relative abundance levels of *Ruminiclostridium-5*. The increased *Bifidobacterium* and *Coprococcus* levels were already noted in the European subjects to be associated with their less severe prediabetic values as compared to the U.S. subjects. Of note, bacteria belonging to *Ruminiclostridium-5* that are strongly reduced in the responders have found to be associated with obesity in children and esophageal cancer in adults, suggesting a role in inflammation.^54,55^

In the present study, we used encapsulated freeze-dried cells of *A. soehngenii*, while in earlier studies with *A. soehngenii* L2–7 we used a liquid formulation. It is likely that the encapsulation improved the potential of ingested viable bacterial cells to reach the intestinal tract. Our earlier work indicated that *A. soehngenii* L2–7 administration can significantly affect small intestinal gene expression and induce GLP-1 production with subsequently improved markers of glucose variability.^26^ This could be a result of lactate depletion and butyrate production, and these all could explain the observed improvement in glycemic control in this large trial with encapsulated freeze-dried cells of *A. soehngenii* CH-106. However, in contrast to pharmaceutical products that consist of a single chemical entity, bacterial cells have a panoply of cellular and metabolic products that may exert health effects. Hence, there are a variety of other mechanistic explanations by which *A. soehngenii* CH-106 could improve cardiometabolic health. Next to butyrate also propionate can be produced as a metabolic end product of *A. soehngenii* CH-106. However, this signaling compound needs the precursor 1,2-propanediol that may be formed from the colonic deoxyhexoses rhamnose or fucose. Another likely possibility is the production of secondary bile salts, as was seen in mouse and man with the isogenic parental strain *A. soehngenii* L2–7.^24–26^ For example, in a recent study with *A. soehngenii* L2–7 in type 2 diabetes patients, an increased level of serum glycoursodeoxycholic acid was observed, and this secondary bile salt has shown to protect mice against insulin resistance.^28^ Similar to *A. soehngenii* L2–7, the genome of *A. soehngenii* CH-106 codes for two bile salt hydrolases and hence it would be of interest to determine the bile salt concentrations of the sera in the present large trial.

Our study has several limitations. First, the trial was performed in sites located both in the U.S. and in Europe. While this improves the generalizability of the study results, participants thus differed in some key parameters according to site, among others that the U.S. participants showed worse metabolic health than the European participants at baseline. Various clinical endpoints were improved most or most significantly in the U.S. site, suggesting that the *A. soehngenii* CH-106 intervention is likely more effective in subjects with more advanced forms of prediabetes. In addition, the sample size of the cohort was relatively modest with close to 100 participants. This precludes powerful statistical outcomes or analysis of the effect of age, gender, ethnicity, or BMI as well as the role of the intestinal microbiota in the clinical response. Another limitation is that the serum HbA1c levels were significantly reduced in the intervention group over placebo but only after a total period of 16 weeks. We attribute this to the long half-time of HbA1c that may thus explain the observed lagged effect. Moreover, in addition to the observed effects on serum HbA1c, the improved glycemic control, and reduced glycemic variability (as determined by CGM analyses) underscores the reduction of glucose excursions over time in the intervention group. Finally, the interventions lasted for 3 months and a longer treatment period may have shown more distinct clinical outcomes.

In conclusion, the results of the current trial with *A. soehngenii* CH-106 confirm and extend our data generated in earlier short-term human intervention studies with the parental *A. soehngenii* strain L2–7.^25^ The intervention with *A. soehngenii* CH-106 in a cohort of U.S. and European prediabetic subjects improves significantly cardiometabolic health. In appreciating this, one should consider that the observed reductions of 1 mmol/mol serum HbA1c and 3 mm systolic blood pressure are relatively small but are found in subjects that are in fact no patients, are reportedly healthy, and receive no medication. What it means is that the intervention with a daily capsule of *A. soehngenii* CH-106 reverts the process of developing serious conditions, such as type 2 diabetes or cardiovascular traumas. Quantitatively larger impact may be expected in patients with type 2 diabetes or hypertension as is supported by the findings of our recent study with the related *A. soehngenii* L2–7 in diagnosed and medicated type 2 diabetes that improved glycemic control and median blood pressure already in 2 weeks.^28^ Hence, further studies include the need to test the *A. soehngenii* CH-106 intervention in larger and more diverse groups of prediabetic subjects as well as in patients with type 2 diabetes or high blood pressure. Finally, the notion that subjects with the highest improvement in HbA1c levels contained the highest relative amounts of gut *Bifidobacterium* spp. would point toward the possibility to combine *A. soehngenii* CH-106 with probiotic *Bifidobacterium* spp. (Figure 5). This would support generating a trophic chain from sugar to lactate and acetate to butyrate, as has been shown previously in simple and complex synthetic communities.^18,20^ All in all, our clinical intervention study provides further support for the daily administration of the butyrate-producing *A. soehngenii* as a next-generation beneficial microbe derived from the human digestive tract to improve the cardio-metabolic health of adults at risk of type 2 diabetes.

## Supplementary Material

Revised_GutMicrobes_Online_Supplemental_Material.docx

## Data and resource availability statement

The datasets generated during and/or analyzed in the current study are available from the corresponding author and guarantor of the work upon reasonable request.
