# Supplementary material for: Anaerobutyricum soehngenii improves glycemic control and other markers of cardio-metabolic health in adults at risk of type 2 diabetes
Source: Gut Microbes. 2025 May 15;17(1):2504115. doi: 10.1080/19490976.2025.2504115 (PMC12087665; doi:10.1080/19490976.2025.2504115)

Online Supplemental Material

# Supplemental Figure 1: CONSORT flow chart

*
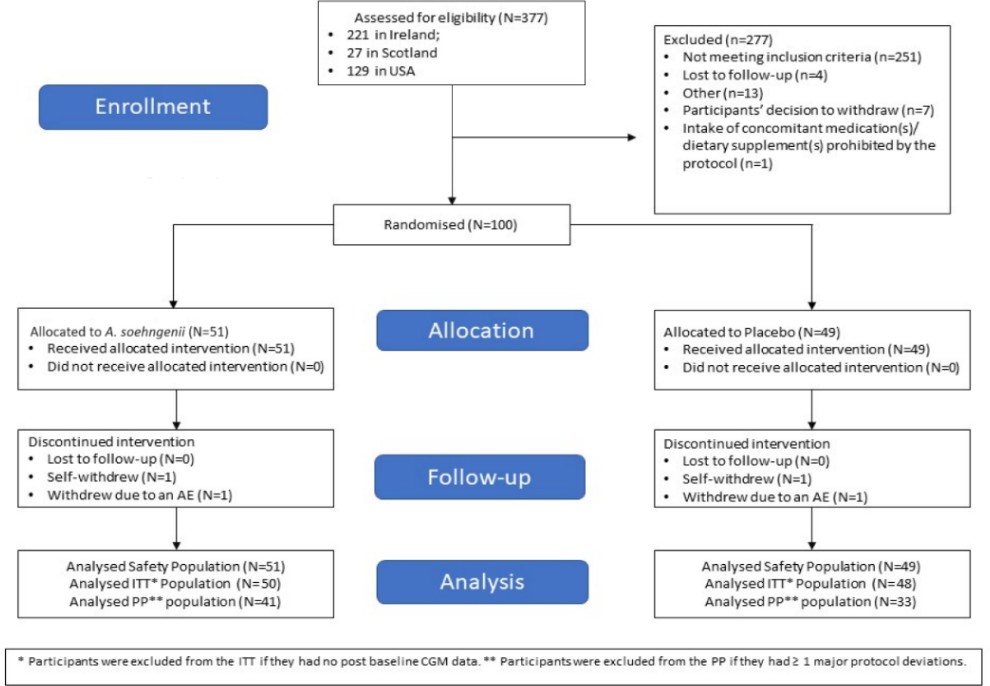
*

*Supplemental Figure 2: Additional endpoints*

**A**


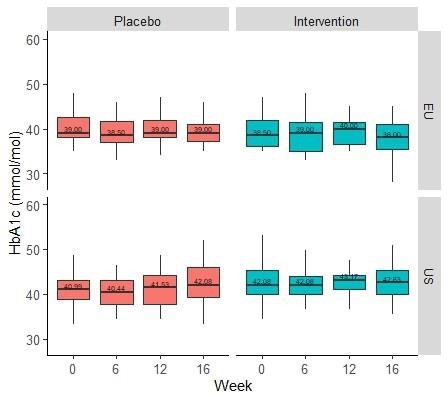

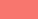

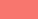

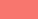

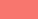

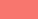

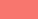

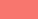

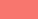

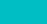

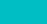

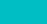

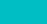

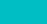

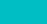

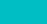

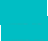


**B**

p=0.233

p=0.050


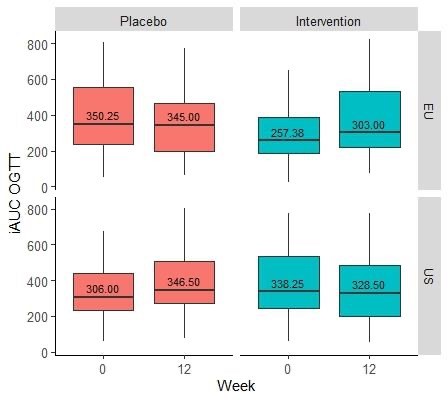

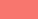

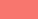

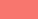

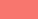

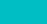

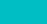

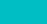

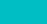


p=0.115

p=0.039

**C**


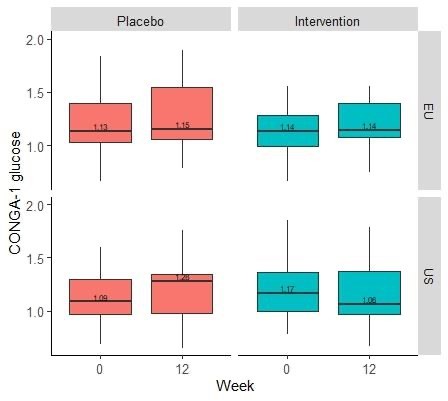

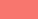

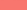

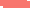

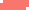

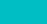

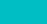

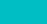

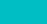


p=0.837

p=0.0323


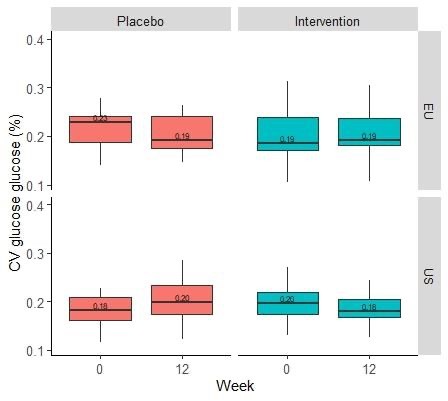

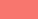

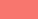

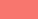

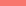

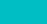

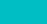

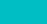

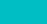


**D**

p=0.011

p=0.0331

**E F**


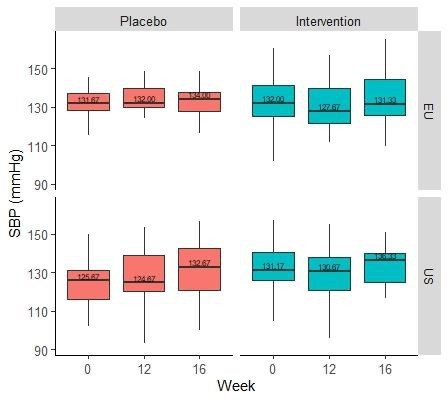

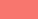

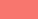

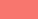

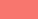

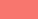

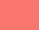

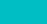

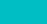

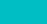

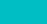

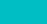

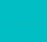


p=0.124

p=0.097


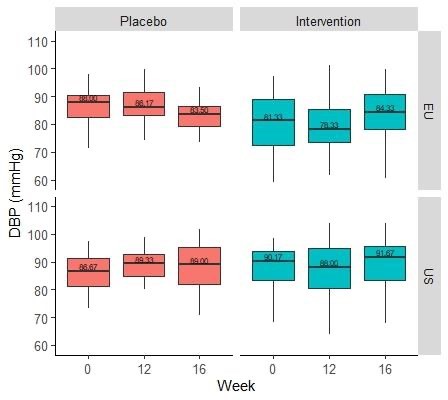

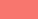

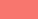

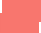

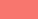

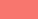

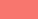

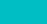

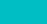

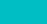

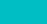

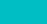

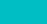

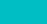


p=0.002

p=0.282

**Supplemental figure 2**: Effect of *A. soehngenii* supplementation on **(A)** iAUC**(B)** HbA1c, **(C)** CONGA-1, **(D)** CV glucose. **(E)** SBP, **(F)** DBP stratified per site center. All analyses performed using a linear mixed effects model on total dataset (lme (y~intervention*week*center). *p*<0.05 was considered statistically significant.

# Supplemental Figure 3: Abundance of A. soehngenii

*
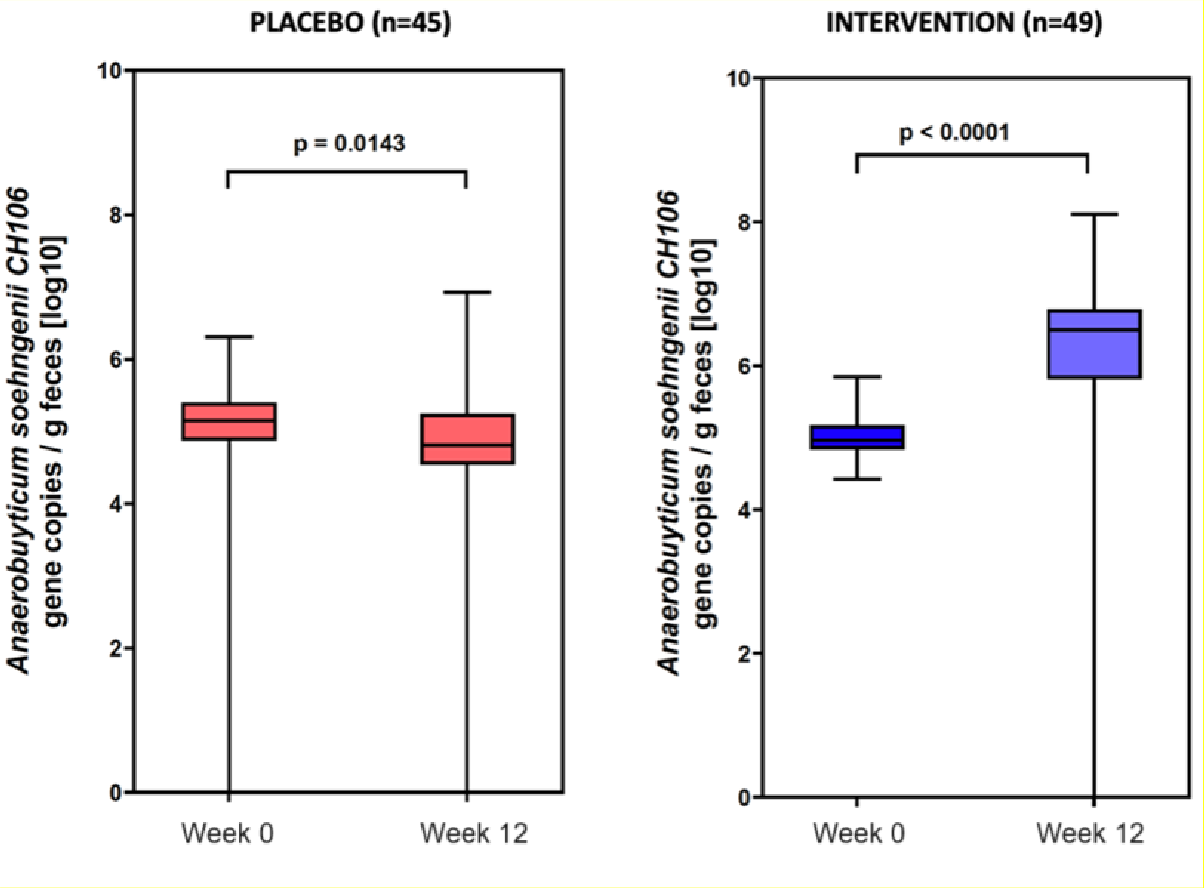
*

Supplemental Figure 3: Absolute abundance of *A. soehngenii* in 16S rRNA copies per g feces of available samples derived from subjects in the placebo red) and *A. soehngenii* (blue) supplementation group at baseline and at week 12 .

# Supplemental Table 1: Study procedures

*
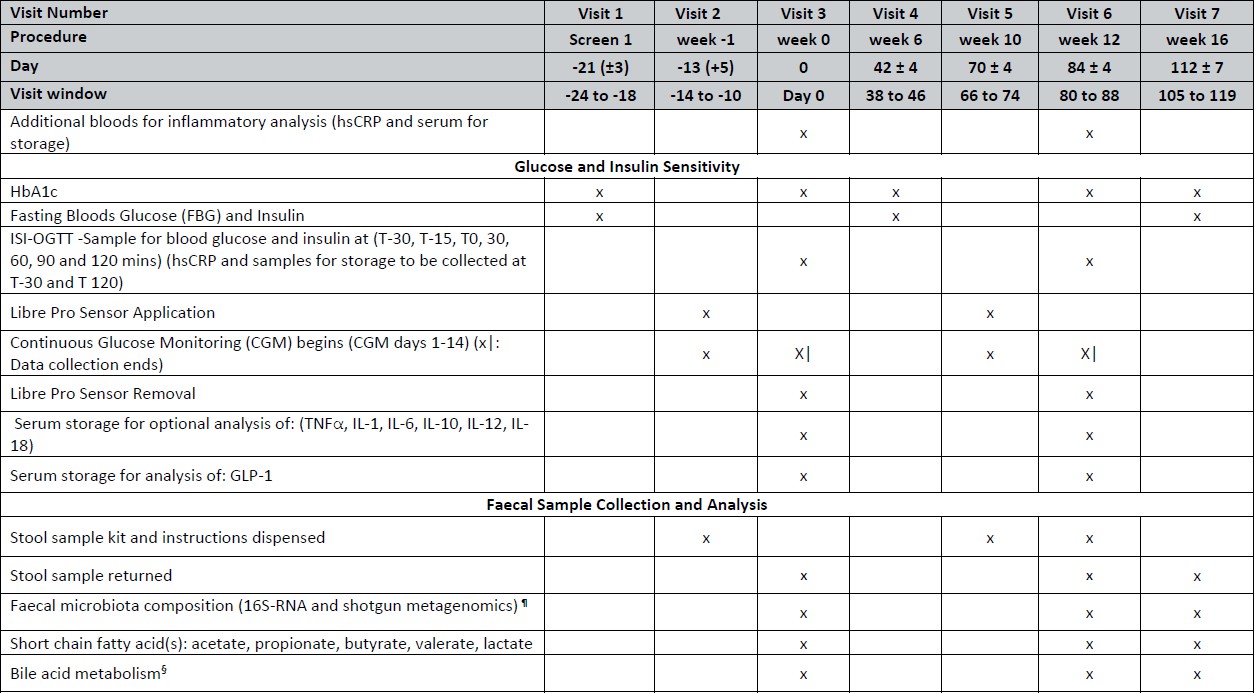
*

*Supplemental Table 2: Changes in endpoints in the total group and per site*

|  | Intervention over Placebo (total) | | Intervention over Placebo per site# | |
| --- | --- | --- | --- | --- |
| Parameter | Change | p-value  for change | Change | p-value for change |
| 2-hr OGTT  iAUC | -39.63 | 0.71 | -57.30 | 0.039 |
| HbA1c*  mmol/mol | -1.0 | 0.031 | -1.09 | 0.050 |
| Coefficient of Variation (CGM) % | -1.0 | 0.013 | -3.1 | 0.033 |
| CONGA-1 (CGM) | -0.06 | 0.045 | -0.20 | 0.032 |
| Diastolic Blood Pressure (mm) | -1.2 | 0.031 | *-3.8* | *0.002* |

*determined over 16 weeks – all other parameters over 12 weeks. #U.S. site underlined and European sites in italics

# Supplemental Table 3: Mean change in safety parameters between baseline and week 12

| Safety parameter* | Unit | *A. soehngenii* | Placebo |
| --- | --- | --- | --- |
| Heart rate | rpm ± s.d. | 0.50 ± 7.6 | -0.83 ± 8.7 |
| Body temperature | ºC ± s.d. | -0.03 ± 0.72 | -0.09 ± 0.47 |
| Blood electrolytes |  |  |  |
| Sodium | mmol/l ± s.d. | -0.05 ± 2.22 | -0.25 ± 2.5 |
| Potassium | mmol/l ± s.d. | -0.08 ± 0.27 | 0.16 ± 0.38 |
| Chloride | mmol/l ± s.d. | -0.16 ± 1.15 | 0.20 ± 1.58 |
| Calcium | mmol/l ± s.d. | 0.05 ± 0.11 | 0.00 ± 0.10 |
| Phosphate | mmol/l ± s.d. | 0.02 ± 0.14 | 0.02 ± 0.13 |
| Magnesium | mmol/l ± s.d. | -0.01 ± 0.06 | 0.00 ± 0.05 |
| Renal profile |  |  |  |
| Urea | mmol/l ± s.d. | -0.02 ± 0.94 | 0.25 ± 0.87 |
| Creatinine | μmol/l ± s.d. | -1.01 ± 8.91 | 1.67 ± 7.46 |
| Uric acid | μmol/l ± s.d. | 11.25 ± 45.8 | -7.85 ± 48.2 |
| Liver function |  |  |  |
| Alanine aminotransferase | IU/L ± s.d. | 1.13 ± 11.1 | -1.56 ± 8.12 |
| Aspartate aminotransferase | IU/L ± s.d. | -0.13 ± 7.82 | 0.07 ± 11.6 |
| Alkaline phosphatase | IU/L ± s.d. | 0.60 ± 9.12 | -0.42 ± 7.37 |
| Gamma‐glutamyl transferase | IU/L ± s.d. | 7.48 ± 21.0 | -0.38 ± 5.99 |
| Total protein | g/l ± s.d. | 0.27 ± 3.52 | 0.07 ± 3.07 |
| Albumin | g/l ± s.d. | 0.56 ± 2.29 | -0.31 ± 2.08 |
| Globulin | g/l ± s.d. | -0.29 ± 2.82 | 0.38 ± 2.25 |
| Total bilirubin | μmol/l ± s.d. | -0.46 ± 3.21 | -0.15 ± 3.71 |
| Complete blood count |  |  |  |
| White Cell Count | 109/l ± s.d. | 0.22 ± 1.49 | -0.09 ± 0.71 |
| Red Cell Count | 1012/l ± s.d. | 0.06 ± 0.21 | -0.01 ± 0.23 |
| Hematocrit | l/l ± s.d. | 0.00 ± 0.02 | 0.00 ± 0.03 |
| Mean Corpuscular Volume | fL ± s.d. | -0.14 ± 1.96 | 0.13 ± 1.91 |
| Mean Corpuscular Hemoglobin | pg ± s.d. | -0.10 ± 0.65 | 0.11 ± 0.58 |
| Mean Corpuscular Hemoglobin Concentration | g/dl ± s.d. | -0.03 ± 0.89 | 0.29 ± 1.72 |
| Red Cell Distribution Width | % ± s.d. | -0.05 ± 0.60 | -0.13 ± 0.44 |
| Platelets | 109/l ± s.d. | 1.91 ± 44.6 | -0.64 ± 22.9 |
| Neutrophils | 109/l ± s.d. | 0.21 ± 1.34 | -0.12 ± 0.63 |
| Lymphocytes | 109/l ± s.d. | 0.01 ± 0.49 | -0.03 ± 0.26 |
| Monocytes | 103/l ± s.d. | 0.00 ± 0.15 | 0.01 ± 0.10 |
| Eosinophils | 109/l ± s.d. | 0.00 ± 0.05 | 0.02 ± 0.14 |
| Basophils | 109/l ± s.d. | 0.00 ± 0.04 | 0.01 ± 0.03 |

*No significant differences were observed between the two groups.

# Supplemental Table 4: Adverse events

| MedDRA Adverse Event Type | *A. soehngenii* | Placebo |
| --- | --- | --- |
| Blood and lymphatic system disorders | 0 | 1 |
| Cardiac disorders | 0 | 1 |
| Ear and labyrinth disorders | 1 | 1 |
| Endocrine disorders | 0 | 2 |
| Gastrointestinal disorders | 13* | 8 |
| General disorders | 3 | 2 |
| Immune system disorders | 1 | 2 |
| Infections and infestations | 6 | 4 |
| Injury, poisoning and procedural complications | 3 | 1 |
| Laboratory out of range values (investigations) | 9 | 8 |
| Metabolism and nutrition disorders | 0 | 1 |
| Musculoskeletal and connective tissue disorders | 6 | 3 |
| Nervous system disorders | 2 | 0 |
| Psychiatric disorders | 1 | 0 |
| Renal and urinary disorders | 1 | 2 |
| Respiratory, thoracic and mediastinal disorders | 2 | 0 |
| Skin and subcutaneous disorders | 2 | 3 |
| Vascular disorders | 0 | 1 |
| Total | 50 | 40 |
| Related to study product | 3 | 3 |

* Abdominal distension, change of bowel habit, diarrhea, flatulence and viral gastroenteritis incidence were numerically but not significantly higher in the *A. soehngenii* supplemented group. No significant differences were observed between the two groups.


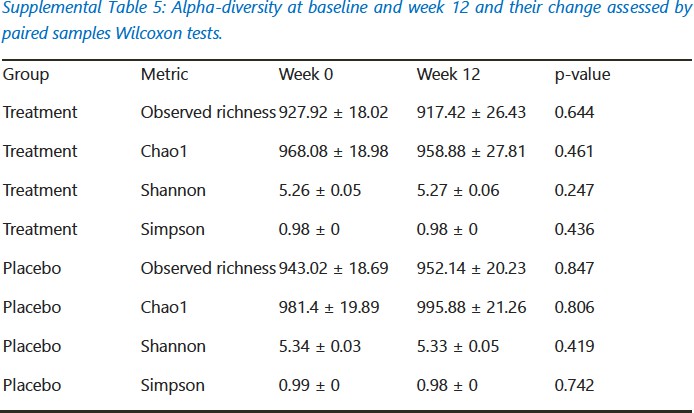

Supplement: Revised_GutMicrobes_Online_Supplemental_Material.docx [file KGMI_A_2504115_SM0836.docx]
